# High-risk human papilloma virus (HPV) and survival in patients with esophageal carcinoma: a pilot study

**DOI:** 10.1186/1471-2407-6-94

**Published:** 2006-04-18

**Authors:** Martin Dreilich, Michael Bergqvist, Martin Moberg, Daniel Brattström, Inger Gustavsson, Stefan Bergström, Alkwin Wanders, Patrik Hesselius, Gunnar Wagenius, Ulf Gyllensten

**Affiliations:** 1 Department of Oncology, University Hospital, 751 85 Uppsala, Sweden; 2 Department of Genetics and Pathology, Rudbeck Laboratory, 751 85 Uppsala University, Uppsala, Sweden

## Abstract

**Background:**

Human papilloma virus (HPV) in patients with esophageal carcinoma has previously been studied with an average detection rate of 15%, but the role of HPV in relation to survival is less clear. In cervical cancer, lung cancer and tonsil cancer HPV viral load is a predictive factor for survival and outcome of treatment. The primary aim was to study the spectrum of high-risk HPV types in esophageal tumors. Secondary, as a pilot study we investigated the association between HPV status and the survival rates.

**Methods:**

We compared both the presence and the viral load of high-risk HPV types 16, 18, 31, 33, 39, 45, 52, 58, and 67 in relation to clinical data from patients with esophageal carcinoma. Survival data and tumor samples were retrieved from 100 patients receiving treatment at the Department of Oncology, Uppsala Hospital, Uppsala, Sweden. The tumor samples were investigated for HPV viral load using real-time PCR.

**Results:**

HPV 16 was detected in 16% of the patients; no other HPV type was detected. HPV 16 infection had no significant effect on survival (p = 0.72). Also, HPV 16 did not improve survival after treatment (radiotherapy or chemotherapy).

**Conclusion:**

Only HPV 16 was detected among the patients. HPV 16 in esophageal carcinoma patients did not influence survival or improve therapy response. However, given the size of the study there is a need to examine a larger cohort in order to understand in more detail the effect of high risk HPV types in esophageal carcinoma.

## Background

High-risk HPV type infections is detected with polymerase chain reaction (PCR) in approximately 15% of esophageal carcinoma patients [1]. The incidence of high-risk HPV type in esophageal carcinoma varies between different geographical areas [1]. It is postulated that areas with high incidence of esophageal carcinoma have higher rates of HPV than in areas with low incidence of esophageal carcinoma [2]. According to epidemiological studies, HPV 16 is the most prevalent HPV type in cervical cancer, followed by HPV 18, 45, 31, and 33, in descending order [3]. HPV infections of squamous cells can induce hyperplasia and papilloma [4] and *in vitro *studies have further revealed that the exposing of human fetal esophageal cells to HPV-18 results in immortalization and development of human esophageal carcinoma cells [5].

In patients with tonsil cancer, the presence of HPV in the tumors correlates with better survival [6]. In a study of patients with cervical carcinoma receiving radiation therapy, HPV-positive patients have a significantly better survival rate [7]. However, within *in vitro *studies the chemosensitivity as well as radiation sensitivity is decreased in HPV-infected cells [8,9]. The immune response might affect survival, but this seems to depend on the whether HPV is present in episomal form or as integrated virus. In patients with cervical carcinomas, higher quantities of antibodies against HPV-16 is seen with episomal HPV as compared to integrated HPV DNA [10]. The mechanisms used by high-risk HPV genotypes (HPV 16, 18, 31, 33, 39, 45, 52, 58 and 67) to overcome the normal cell cycle control is mediated by the HPV viral oncoproteins E6 and E7, which cause inactivation of the tumor suppressor genes p53 and Rb [11].

In the present study, viral load in a series of high-risk HPV types (16, 18, 31, 33, 39, 45, 52, 58 and 67) were determined in esophageal carcinoma tumors. Secondary, as a pilot study we investigated the association between HPV status and survival in patients with esophageal carcinoma.

## Methods

### Ethics

The study was reviewed and approved by the research ethics committee, Uppsala University, Uppsala, Sweden.

### Patients characteristics

Patients with esophageal carcinoma, admitted to the Department of Oncology, University Hospital, Uppsala, Sweden, during 1990–2000 were identified. These patients formed a consecutive series. A total of 126 patients fulfilled the inclusion criteria but 26 patients were lost due to inadequate (n = 9) or absence of histological material (n = 17). The present study is based on a retrospective examination of esophageal carcinoma diagnostic biopsy samples from clinical cases, all retrieved tumor samples were re-evaluated before analysis.

The following parameters were studied: age, gender, weight loss, performance status according to the Eastern Cooperative Oncology Group (ECOG) [12] at first admittance, smoking habits, alcohol habits and differentiation of the tumor. Anatomical localization of the tumor were grouped into an upper part (15–24 cm), a middle part (25–34 cm) and a lower part of the esophagus (35–46 cm. The tumor status were characterized into localized (Primary tumor with or without local node metastases) or advanced disease (With distant metastases).

Treatment strategy was recorded as:

1. Preoperative radiation treatment, total dose of 40 Gy in 2 Gy fractions. This irradiation was given concomitant with chemotherapy (Cisplatin 100 mg/m^2 ^and 5-FU 750 mg/m^2^, three cycles with an interval of three weeks of which two were given concomitantly).

2. Curatively intended radiation treatment, total doses 60–64 Gy.

3. Palliative treatment included radiation treatment, total dose 36 Gy in 3 Gy fractions. These patients also received brachytherapy and in some cases, also palliative chemotherapy.

4. Palliative treatment only including chemotherapy.

### HPV plasmids

Plasmids containing HPV 16, 18, 31, 33, 39, 45, 52, 58 and 67 were supplied by T. Matsukura (National Institute of Health, Japan), A. Lörincz (Digene Corporation) or G. Orth (Institut Pasteur, Paris) or prepared by cloning from PCR products of clinical samples. The plasmids were used both as positive controls and to estimate the sensitivity of the assay.

### Deparaffination procedure

The biopsies were fixated by treatment in buffered formalin followed by paraffin embedding. Paraffin blocks from the primary tumor were cut in 10-μm sections and 4 sections/patient were collected in the same microcentrifuge tube. Samples were de-waxed in 1 ml xylene for 5 min. in room temperature and centrifuged at 14,000 rpm for 5 min. The supernatant was removed. This step was then repeated 5 times. Samples were gently vortexed between each step. 1 ml 95% ethanol was then added for 5 min. in room temperature and this was repeated with 70% ethanol. The samples were centrifuged 14.000 rpm for 5 min. between the ethanol changes. The samples were then dried in a 37°C heated block with open lids for 20 min. to remove residual ethanol.

### Proteinase K digestion

200–400 μl of dilution buffer was added to each tube (0.2 M Tris-HCL pH 8.0, 1% sodium dodecyl sulfate, 10 mM EDTA, 1 mg/ml proteinase K). The volume of the dilution buffer was dependent of the amount of tissue available. Samples were subsequently incubated at 50°C overnight and thereafter heated to 94°C for 10 min. to inactivate the proteinase K.

### Protein precipitation

50 μl of saturated NaCl solution (approximately 6 M) was added to each tube and the samples gently vortexed for 5 min. The tubes were then centrifuged at 14.000 rpm at room temperature for 5 min. After centrifugation, white pellets were visible in the bottom of the tubes and supernatants were transferred to a new tube.

### DNA precipitation

DNA was precipitated with 2.5 volumes of 95% ethanol in -20°C for 1 hour and then pelleted by centrifugation at 14.000 rpm in room temperature for 30 min. The pellet was then washed with 70% ethanol followed by centrifugation for 5 min. DNA pellets were air dried and finally re-suspended in 100 μl TE buffer (100 mM Tris-HCL, pH8.0, 1 mM EDTA).

### Real-time PCR

The detection of the HPV was performed using a real-time PCR based method previously described [13]. Briefly, the real-time PCR assay detects and quantifies HPV 16, 18, 31, 33, 39, 45, 52, 58 and 67. The assay is based on three parallel real-time PCRs from each patient sample: a) Reaction 1 detects and quantifies HPV types 16, 31, 18 and 45 (HPV 18 and 45 detected and quantified together) using three different fluorophores, b) Reaction 2 detects and quantifies HPV types 33, 39, 52, 58 and 67 (HPV 33, 52, 58 and 67 detected and quantified together), again using three different fluorophores, and c) Reaction 3 detects and quantifies the amount of a human single copy gene (HMBS, Homo sapiens hydroxymethylbilane synthase, GenBank accession number M95623.1). Reaction 1 includes a total of seven PCR primers and three probes, Reaction 2 a total of five PCR primers and two probes and Reaction 3, two PCR primers and a single probe. All probe and primer sequences have been described [13]. By relating the HPV copy number to the number of nuclear gene equivalents (from Reaction 3) a measure of HPV load is obtained.

The system has a dynamic range from 10^2 ^to 10^7 ^HPV copies per assay and is applicable to both fresh clinical samples and DNA extracted from archival samples. Reconstitution experiments, made to mimic infections with several HPV types, shows that individual HPV types can be detected in a mixture as long as they represent 1–10 percent of the main type. The system has been evaluated with respect to technical specificity and sensitivity, reproducibility, reagents stability, sample preparation protocol and applied to the analysis of clinical samples. This homogeneous assay provides a fast and sensitive way for estimating the viral load of a series of the most frequent oncogenic HPV types in biopsies.

The PCR amplification was performed in a 25 μl volume containing 1× PCR buffer gold (Applied Biosystems, Foster City, CA, USA), 300 μM 6-carboxy-X-rodamine (Molecular Probes inc, Eugene OR, USA), 3.5 mM MgCl_2_, 200 nM each of dATP, dCTP, dGTP and 400 nM dUTP (Pharmacia Biotech, Uppsala, Sweden), 0.625 U AmpliTaq Gold (Applied Biosystems, Foster City, CA, USA), 3 μg BSA (Sigma Chemical Co., St. Louis, MO, USA) and 200 nM of each primer and probe, and 3 μl DNA extract. Amplification and detection was performed using a 7700 Sequence Detection System (Applied Biosystems Inc., Foster City, CA, USA). The amplification ramp included an initial hold step of 10 min. at 95°C followed by a two-step cycle consisting of 15 sec. at 95°C and 1 min. at 57°C, repeated 40 times. Each real-time PCR run included reactions with no template controls containing all PCR components but without template DNA to ensure that the reagents mix were free of contaminants.

### Statistics

The survival rates were estimated with the Kaplan-Meier product limit method, whereas univariate analysis was performed with log-rank test. Cox regression analysis was performed to investigate if certain continuous factors had a significant effect on survival. Spearman's rank order correlation was utilized for tests of associations between factors. A 5% significance level was used throughout the study.

## Results

A total of 100 patients with esophageal carcinoma were investigated for the presence of the following types of human papilloma virus 16, 18, 31, 33, 39, 45, 52, 58, and 67. The patients were followed until 2003 with a median follow up time of 297 days (5–3675 days) and 11 (11%) patients were alive after follow-up. Patient's characteristic, median survival and median viral load for each investigated parameter are listed in Table 1. Univariate analysis revealed that tumor stage (p = 0.00071) and performance status according to ECOG (p < 0.0001) were associated with survival. HPV positive patients characteristics are listed in Table 2 including viral load, gender, performance status, stage of disease (localized or with metastases), treatment approach and survival time.

**Figure 1:**
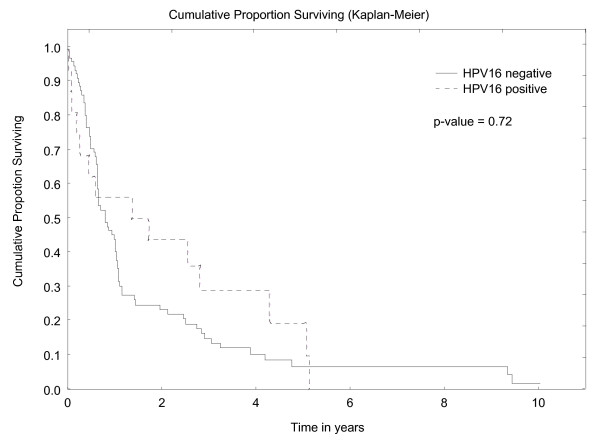
Survival analysis including all patients based on detection of amplified HPV 16 DNA in the tumor samples.

**Figure 2:**
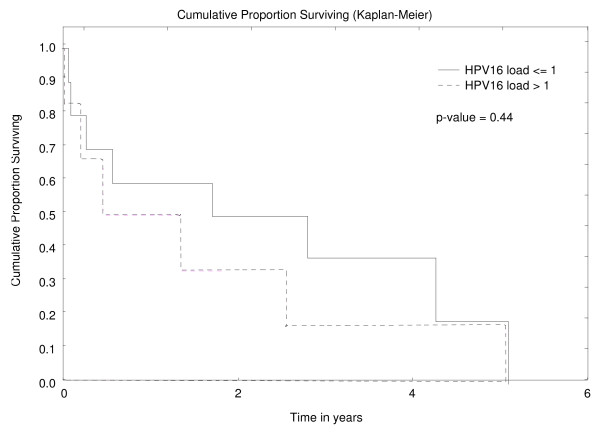
Survival analysis including sixteen HPV 16 positive cases with respect to their HPV 16 viral load, divided into HPV 16 load <1.0 genomes/human genome equivalents or HPV 16 load >1.0 genomes/human genome equivalents.

Prior to the analysis of clinical samples, standard curves for the different HPV types were generated based on dilution series of the HPV plasmids, as described [13]. Each run of samples included negative controls (no PCR template) and positive controls (HPV plasmid in low copy number). In none of the real-time runs did the controls yield a different signal than that expected. Reaction 3 (containing the reagents for the nuclear single copy gene HMBS) was used to evaluate the DNA quality. Only samples with positive reaction was further investigated for HPV type. The HMBS assay was positive, i.e. the reactions contained DNA suitable for PCR, in all tumors examined. Amplification of HPV 16 DNA was detected in 16% of the patients and no other HPV type was detected. HPV 16 status did not influence survival rate (p = 0.72) (Figure 1). The HPV 16 viral load varied between samples with a median HPV 16 viral load of 4.3 genomes/human genome equivalents, but no correlation with survival rate was detected (p = 0.44) (Figure 2). Histological subgroups did not have a difference in survival depending on HPV 16 status, either in patients with squamous cell carcinoma (p = 0.61) or adenocarcinoma (p = 0.49). Finally, HPV 16 status did not have any effect on survival after radiation treatment (p = 0.16) or chemotherapy (p = 0.22).

## Discussion

HPV is an important risk factor for development of cervical cancer as well as head/neck and anal cancer [14-17]. In the present study we investigated the presence and viral load of a series of high-risk HPV genotypes in 100 patients with esophageal carcinoma using real-time PCR. Tonsil cancer as well as tumors located in the oropharynx and hypopharynx have high rates of HPV positive cases. In tonsil and oropharyngeal tumors approximately half of all cases harbor detectable HPV, whereas in tumors in the oral cavity and hypopharynx HPV is detected in approximately 20% of all cases. According to earlier studies, HPV 16 is the most common type of HPV associated with head and neck malignancies, while other high-risk HPV types are rare [13,14]. Smoking and excessive alcohol consumption is considered risk factors for both squamous cell carcinomas and tonsil cancer. However, a subgroup of younger, no smoking tonsil cancer patients with HPV positive tumors indicates a different etiology for tonsil cancer besides excess smoking and alcohol consumption [6,13]. In the present study, we were unable to address this because of few observations in the HPV positive group of patients.

Viral load has previously been investigated in esophageal carcinoma tumors with PCR technology [18]. Si et al report up to 157 genomes/human genome equivalents for HPV 16 in esophageal carcinoma tumors. By contrast, HPV 18 was only detected in 6 of 319 cases and a median HPV viral load of 4.9 genomes/human genome equivalents [18]. Weston et al differentiates the HPV types as high risk and low risk HPV types and report one of 40 cases with a high risk HPV type [19]. In our study, HPV 16 was detected in 16% of the cases, but none of the other viral types were found.

The prognostic value of HPV status has previously been investigated in patients with esophageal carcinoma [20,21]. Furihata et al report that HPV positive patients have worse survival than HPV negative patients with an over-expression of p53 in esophageal carcinoma patients. They conclude that each of the two factors, HPV infection and p53 over-expression, indicate poor prognosis [21]. Hippelainen et al examined 61 patients with esophageal carcinomas and report that HPV were involved in 11% of the tumors but without prognostic value for presence of HPV [20]. In the present study, HPV 16 infection was not associated with higher survival rate. However, for patients having a HPV 16 viral load > 1.0 viral genome per cell, an indication of higher survival rate was found in comparison with patients having a HPV 16 viral load < 1.0 viral genome per cell (Figure 2).

Lindel et al report HPV status as a prognostic parameter for radiotherapy response in cervical carcinoma patients [22]. Further, Harima et al report that HPV negative patients have poorer outcome than HPV positive patients after radiotherapy and concludes that HPV status might be useful to optimize for treatment options [7].

We investigated the HPV positive patients in relation to treatment. HPV positive patients receiving radiation treatment indicated a trend towards better survival, although this was not statistically significant (p = 0.16). Integration of HPV result in higher expression of the oncoproteins E6/E7, thereby abrogating the p53 and Rb protein functions, promoting genomic rearrangements [23] and development of cervical carcinoma [24]. Rearranged DNA is theoretically more sensitive to radiation, providing an explanation for the indication of a higher survival rate for patients with HPV positive tumors. However, our results should be interpreted with caution due to the small number of cases investigated. For patients receiving chemotherapeutic treatment (mainly neoadjuvant cisplatin-based chemotherapeutic combinations), we see a tendency towards better survival (p = 0.22). *In vitro *studies report that cisplatin sensitivity is increased in HPV 16 transfected ovarian cancer cells [25], in accordance with results from the present study.

In conclusion, we employed a sensitive real-time PCR assay for analysis of paraffin-embedded tissue from patients with esophageal carcinoma. Among the 100 patients investigated, HPV 16 was present in 16% of the patients. Given the limited sample size, the results of this pilot study should be interpreted with caution. However, the results indicate a positive association of HPV 16 viral load with survival and motivate further studies of the role of HPV 16 viral load in patients with esophageal carcinoma.

## Conclusion

Only HPV 16 was detected among the patients. HPV 16 in esophageal carcinoma patients did not influence survival or improve therapy response. However, given the size of the study there is a need to examine a larger cohort in order to understand in more detail the effect of high risk HPV types in esophageal carcinoma.

## Competing interests

The author(s) declare that they have no competing interests.

## Authors' contributions

MD and MB contributed equally as principal investigators and UG was the principal supervisor. SB, DB, GW assisted in the design of the study and obtaining funding. PH was responsible for the statistical analyses. AW, MM and IG contributed with evaluation of the material and did the laboratory analyses. All these investigators contributed to data interpretation and preparation of the paper.

## Pre-publication history

The pre-publication history for this paper can be accessed here:



## Figures and Tables

**Table 1:** Demographic description of patients for all investigated parameters. The distribution of HPV 16 positivity and median viral load is shown for all clinical subgroups. Tumor stage and performance status according to ECOG were correlated to patients survival in the univariate survival analysis, p-value is shown for all parameters. *Treatment management not included in survival analysis.

	Number of patients	Median survival in days	Univariate survival analysis p-value for each parameter	HPV 16 pos	HPV 16 viral load median value based on positive samples (genomes/human genome equivalents)
**Gender**			0.55		
Male	71	292		10	2.7
Female	29	338		6	10.5
**Age**			0.84		
65	39	292		7	2.1
65 +	61	307		9	6.6
**Tumour stage**			0.00055		
Advanced disease	30	212		5	2.12
Localised disease	70	376		11	5.3
**Histology**			0.39		
Squamous cell carcinoma	71	250		10	16.4
Adenocarcinoma	27	374		5	0.6
Mucoepidermoid	2	528		1	5.3
**Tumor differentiation**			0.46		
Low differentiated	29	362		4	1.4
Middle	23	234		4	1.9
High	2	187		0	-
Not determined	46				
**Localisation of tumor**			0.17		
Upper	11	292		3	15.7
Middle	33	338		6	16.9
Lower	47	235		3	0.16
Not determined	9				
**Performance status**			<0.0001		
0	43	419		6	2.7
1	43	235		4	16.3
2	8	120		2	60.6
3	4	49		3	0.6
Not determined	2				
**Weight**			0.12		
Weight loss	82	236		10	0.6
No weight loss	18	414		6	6.0
**Smoking habits**			0.45		
Non smoker	16	309		1	5.3
Smoker	45	292		4	8.7
Ex-smokers	22	235		6	2.7
Not determined	17				
**Alcohol usage**			0.81		
Alcohol use	50	339		6	5
No alcoholic use	6	450		1	5
Not determined	44				
*Treatment*			*		
Surgery+ Chemo-radiation	13	493		1	0.5
Curativ intended Chemo-radiation	24	445		5	5,3
Palliative chemotherapy and radiotherapy	47	234		5	15,7
Palliative chemotherapy	16	125		5	0.6

**Table 2:** Description of HPV positive patients characteristics including viral load, gender, histology, performance status, stage of disease, survival days and treatment strategy. *genomes/human genome equivalents. **alive at follow up, censored in the survival analyze. Nd-the parameter could not be determined. Histology: ESCC-Squamous cell carcinoma, AC-Adenocarcinoma, MEC-Mucoepidermoid carcinoma. Treatment strategy: 1. Pre-operative concommitant chemoradiotherapy, 2. Curatively intended radiotherapy 2–60 Gy, 3. Palliative intended radiotherapy and chemotherapy, 4. Palliative care including chemotherapy.

HPV16 Viral load*	**Gender**	**Histology**	**Performace status**	**Advanced disease**	**Survival days**	**Treatment strategy**
437,0	Male	ESCC	3	No	23	3
< 94	Women	ESCC	2	Yes	207	2
<31	Male	ESCC	1	No	98	3
27,3	Male	ESCC	2	Yes	30	4
<17	Women	ESCC	1	No	1569	2
15,7	Women	ESCC	1	No	626	3
6,6	Male	ESCC	0	No	**1343	3
5,3	Women	MEC	0	No	650	2

3,3	Male	ESCC	0	No	1028	2
2,1	Male	AC	0	Yes	1870	4
0,6	Male	AC	nd	No	1854	3
0,6	Women	AC	3	No	73	4
0,5	Male	ESCC	0	No	492	1
0,2	Male	AC	0	No	934	2
0,2	Women	AC	1	Yes	168	4
0,1	Male	ESCC	3	Yes	6	4
